# 108 An Introductory Systematic Review of the Vancouver Scar Scale: Versions, Validations and Utilizations

**DOI:** 10.1093/jbcr/iraf019.108

**Published:** 2025-04-01

**Authors:** Deborah Knight, Jennifer Parks

**Affiliations:** Joseph M. Still Burn Center at Doctors Hospital; HCA Healthcare

## Abstract

**Introduction:**

Since its development in 1990, the Vancouver Scar Scale (VSS) has been validated and modified in order to objectively rate the appearance of a burn scar. The VSS subjectively assesses the pigmentation, vascularity, pliability, and height of the burn scar and was the first scale that attempted to capture these components of maturing burn scars. In 2024, this tool is still considered one of the most common evaluation methods for scars despite low inter-rater reliability, inconsistent validity, and multiple modifications. A systematic review was completed to assess VSS and its modifications, as well as current trends in its utilization.

**Methods:**

A PRISMA-compliant qualitative systematic review was conducted on studies published since 1990 making reference to the VSS. One thousand twenty seven studies were provided as a result of the search. Due to the volume of abstracts found, this introductory review was further limited to then include only the 107 studies published between January 1, 2024 and September 30, 2024.

**Results:**

Of the 107 studies reviewed, only 34 were excluded due to lack of full text availability, two of which were not available in English. Majority of studies were assessing non-burn scars, did not specify the parameters of the VSS, nor provide any explanation of the assessment. Only 5 of the included 73 studies comprehensively interpreted the VSS outcomes and only 3 acknowledged the weakness of the VSS as a comprehensive scar assessment. Several articles added assessment components such as pain and pruritus while others adjusted the scoring system for the tool. Nearly half of the publications reviewed did not provide a reference to indicate which version of the VSS was being utilized. Majority of the included studies were published in peer-reviewed journals which fall in the domains of Plastic Surgery, Dermatology, and Orthopedics.

**Conclusions:**

The VSS has been validated for use with burn scars, yet its current utilization in published literature falls outside of burn-specific populations, while its limitations and poor inter-rater reliability are not fully acknowledged in these publications. Furthermore, the insufficient documentation referencing the specific version of the VSS utilized brings forth concerns for validity and credibility of some published studies. Further review is required to fully assess the progression from the original validation of the VSS to its present day general utilization.

**Applicability of Research to Practice:**

To the extent that the VSS is being utilized in practice, research, and publications, this study reinforces the need for further research to validate the credibility of the VSS and strengthen the impact of associated publications

**Funding for the Study:**

No external funding was provided

